# Quantifying the Digital Traces of *Hurricane Sandy* on *Flickr*

**DOI:** 10.1038/srep03141

**Published:** 2013-11-05

**Authors:** Tobias Preis, Helen Susannah Moat, Steven R. Bishop, Philip Treleaven, H. Eugene Stanley

**Affiliations:** 1 Warwick Business School, University of Warwick, Scarman Road, Coventry, CV4 7AL, UK; 2 Department of Physics, Boston University, 590 Commonwealth Avenue, Boston, Massachusetts 02215, USA; 3 Department of Mathematics, UCL, Gower Street, London, WC1E 6BT, UK; 4 Department of Computer Science, UCL, Gower Street, London, WC1E 6BT, UK; 5 These authors contributed equally to this work.

## Abstract

Society’s increasing interactions with technology are creating extensive “digital traces” of our collective human behavior. These new data sources are fuelling the rapid development of the new field of computational social science. To investigate user attention to the *Hurricane Sandy* disaster in 2012, we analyze data from *Flickr*, a popular website for sharing personal photographs. In this case study, we find that the number of photos taken and subsequently uploaded to *Flickr* with titles, descriptions or tags related to *Hurricane Sandy* bears a striking correlation to the atmospheric pressure in the US state New Jersey during this period. Appropriate leverage of such information could be useful to policy makers and others charged with emergency crisis management.

Steadily increasing quantities of data are being generated through society’s interactions with technology, automatically documenting human actions in a previously unimaginable fashion^1^,^2^,^3^,^4^,^5^,^6^,^7^,^8^,^9^,^10^. Analysis of such “big data” is opening up new windows for a more precise quantification of real world social phenomena. A particularly fruitful area of research has focused on the analysis of Internet user search queries, as logged by search engines such as *Google*. Strong links have been found between changes in the information users are seeking online and events in the real world, ranging from reports of flu infections across the USA^11^ to trading volume in the US stock markets^12^. A recent study has also shown that Internet users from countries with a higher per capita GDP are significantly more likely to search for information about years in the future than years in the past^13^. Preis, Moat and Stanley have demonstrated that changes in the number of searches for financially related terms on *Google* may have contained early warning signs of stock market moves^14^. Moat *et al.* presented evidence that increases in the number of views of financially related pages on *Wikipedia* could be detected before stock market falls^15^.

Collective human attention to topics can be measured by various indices into online information flow. While analysis of search volume provides insight into the information that people are seeking, there are other data sources which one can analyze to gain insight into information that people are distributing. This information can take various forms, from text to multimedia, such as photos and videos.

In this case study, we analyze the usage of a prominent photo sharing website, *Flickr*^19^,^20^,^16^,^17^,^18^. We investigate whether we can identify any relationship between catastrophic events such as natural disasters, and users’ photo sharing activity on *Flickr,* to provide insight into the dynamics of human attention to such events.

The subject of our case study is *Hurricane Sandy*—a hurricane that devastated portions of the Caribbean and the Mid-Atlantic and Northeastern United States during late October 2012. “Sandy”, classified as the eighteenth named storm and tenth hurricane of the 2012 Atlantic hurricane season, made landfall near Atlantic City, New Jersey at 00:00 *Coordinated Universal Time* (UTC) on 30 October 2012^21^.

## Results

**Figure 1:**
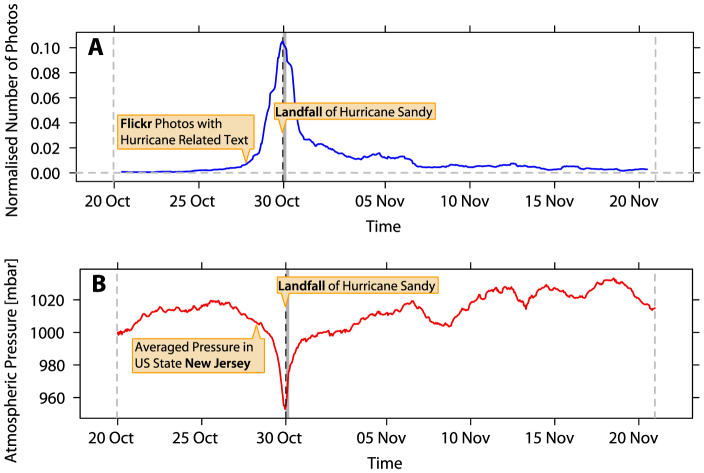
Hurricane Sandy related *Flickr* photos and atmospheric pressure in the US state New Jersey. (A) We identify all photos taken between 20 October 2012 and 20 November 2012 which were subsequently uploaded to *Flickr* with any of the three terms *Hurricane*, *Sandy* and *Hurricane Sandy* in their tags, title or description text. Here we show the number of these Hurricane Sandy related *Flickr* photos normalized by the total number of photos taken and subsequently uploaded to *Flickr*. The data are analyzed at an hourly granularity. To eliminate daily periodicity in the hourly *Flickr* data, the data are transformed to represent the average value from a moving window spanning 24 hours (Δ*t* = 24 hours). Date lines denote the beginning of a day in UTC. (B) The atmospheric pressure in New Jersey between 20 October 2012 and 20 November 2012. Atmospheric pressure data is compiled from average measurements from 62 stations in New Jersey that form part of the *Automated Surface Observing System* (ASOS). Again, the data are analyzed at an hourly granularity.

We examine photos uploaded to *Flickr* and labeled with the terms *Hurricane*, *Sandy* or *Hurricane Sandy* in their tags, title or description text. We analyze the times at which these photos were taken by users around the world. We normalize hourly counts of photos labeled with these hurricane related terms by the hourly count of all photos taken. To eliminate daily periodicity in the *Flickr* data, the counts for photos labeled with the terms *Hurricane*, *Sandy*, *Hurricane Sandy*, and for all photos taken are transformed to represent at each hour *t* the average value from a surrounding moving window of Δ*t* hours (*t* − Δ*t*/2; *t* + Δ*t*/2]. Visualization of the data reveals that the normalized number of photos taken increased continuously while “Sandy” was moving toward the coast of the United States (Figure 1A).

In this case study, we compare the normalized number of *Hurricane Sandy* related *Flickr* photos taken to a direct measure of the environment during the development of *Hurricane Sandy*: the atmospheric pressure in the US state New Jersey between 20 October 2012 and 20 November 2012 (Figure 1B). Atmospheric pressure data are compiled from average measurements from 62 stations in New Jersey forming part of the *Automated Surface Observing System* (ASOS), and are analyzed at an hourly granularity.

We find a striking correlation between the moving average of the normalized number of *Hurricane Sandy* related *Flickr* photos taken and the atmospheric pressure in New Jersey for Δ*t* = 24 hours (Kendall’s tau = −0.37, *z* = −15.14, *p* < 0.001). Notably, the time of landfall of *Hurricane Sandy* not only marks the time of lowest air pressure, but also the time at which the largest number of *Flickr* photos labeled with terms related to *Hurricane Sandy* were taken. We find qualitatively similar results for a moving average window with Δ*t* = 12 hours (Kendall’s tau = −0.36, *z* = −14.62, *p* < 0.001). Analysis of the interval starting 48 hours before and ending 48 hours after landfall of *Hurricane Sandy* also reveals qualitatively similar results for Δ*t* = 24 hours (Kendall’s tau = −0.83, *z* = −12.02, *p* < 0.001) and Δ*t* = 12 hours (Kendall’s tau = −0.73, *z* = −10.63, *p* < 0.001).

## Discussion

In summary, the number of photos taken and subsequently uploaded to *Flickr* with labels related to *Hurricane Sandy* bears a striking correlation to the atmospheric pressure in the US state New Jersey in the period from 20^th^ October 2012 until 20^th^ November 2012. We propose two possible interpretations of this result. First, we suggest that users may have taken more photos as the severity of the problem increased – in this case, atmospheric pressure dropping and therefore wind speed increasing. This would suggest that in cases where no external sensors were available, it may be possible to measure the number of *Flickr* photos relating to a topic to gauge the current level of this category of problems. A second alternative interpretation would be that users were well informed as to the expected time of landfall due to extensive media coverage, and that their attention to the problem increased as the anticipated climax of the disaster approached, leading to an increase in the numbers of photos taken. This would equally open the possibility that increases in *Flickr* photo counts with particular labels may reveal notable increases in attention to an issue, such that issues which have received less extensive media coverage but which may merit further investigation may be identified. Future research investigating other examples of catastrophic events would be needed to demonstrate universality of the results we find. Such research should also take into account the number of active *Flickr* users in a country, given the country’s population.

We suggest that *Flickr* can be considered as a system of large scale real-time sensors documenting collective human attention. The analysis of other examples of catastrophic events, beyond this case study of Hurricane Sandy, is however needed to evaluate whether an appropriate leverage of such a system could be of interest to policy makers and others charged with emergency crisis management.

## Methods

We retrieved data on image uploads to *Flickr* by accessing the *Flickr* API (http://www.flickr.com/services/api/flickr.photos.search.html) on 3 December 2012. The photo search function used returns a list of photos matching given criteria. We retrieved data on atmospheric pressure from 62 weather stations in New Jersey which form part of the *Automated Surface Observing System* (http://www.ncdc.noaa.gov/land-based-station-data/automated-surface-observing-system-asos) on 28 December 2012.

## Author Contributions

T.P., H.S.M., S.R.B., P.T. and H.E.S. performed analyses, discussed the results, and contributed to the text of the manuscript.
